# Prognostic value of modified KDIGO staging for acute kidney injury in neonates: a prospective observational study in a level IIIB NICU

**DOI:** 10.1186/s12887-025-06457-z

**Published:** 2026-01-20

**Authors:** Praveen C Samuel, Vijay Bhaskar Reddy Badduri, Juanitha George, Leslie E Lewis, Jayashree Purkayastha

**Affiliations:** Department of Pediatrics, Kasturba Medical College, Manipal Academy of Higher Education, Manipal, Karnataka India

**Keywords:** Acute kidney injury (AKI), Neonatal intensive care unit (NICU), Modified KDIGO, Sepsis, Neonatal mortality, Inotropic support, Nephrotoxic exposure, Growth recovery, Neonatal outcomes, Shock, Congenital heart disease, Neonatal intensive care

## Abstract

**Background:**

Acute kidney injury (AKI) is an increasingly recognized complication in neonates admitted to intensive care, contributing significantly to short-term morbidity and mortality. Early diagnosis and risk stratification using standardized criteria such as modified neonatal Kidney Disease Improving Global Outcomes (KDIGO) remain critical, particularly in resource-constrained tertiary units. Limited Indian studies have evaluated AKI across both term and preterm neonates in Level IIIB Neonatal Intensive Care Unit (NICU) with post-discharge growth follow-up.

**Methods:**

This prospective observational study was conducted over one year (2023–2024) in a Level IIIB NICU. Renal-function tests were performed in 645 of 925 admissions (69.7%), and all neonates fulfilling modified neonatal KDIGO criteria were included. Demographic, clinical, biochemical, and therapeutic variables were analyzed; follow-up anthropometry was assessed six weeks after discharge. Multivariate regression identified independent predictors of AKI severity and mortality.

**Results:**

Among 925 admissions, 53 neonates met inclusion criteria for AKI, giving an incidence of 8.2%. Term neonates (56.6%) were more frequently affected than preterm. Stage 3 AKI carried the highest mortality (90%, *p* = 0.032). Sepsis (59%), shock (58%), and congenital heart disease (53%) were the major contributors. Inotropic support was required in 58.5% of cases. Nephrotoxic exposure occurred in 62%, predominantly to amikacin. Non-oliguric AKI was more common (70%). Post-discharge assessment showed that preterm small-for-gestational-age (SGA) infants achieved the greatest catch-up growth (38.5 g/day weight gain, 5 cm/month length velocity). The newly developed AKI risk score demonstrated an AUC of 0.81 (95% CI 0.74–0.88) for predicting severe AKI.

**Conclusion:**

The incidence of AKI in this Level IIIB NICU was 8.2%, with sepsis, shock, and congenital heart disease as predominant causes. The modified KDIGO criteria enabled early detection and accurate staging. Follow-up growth trends and quantified nephrotoxic exposure provide perspectives seldom addressed in prior Indian studies. Regular renal monitoring and risk-adjusted supportive care, coupled with longitudinal follow-up, are essential to improve neonatal outcomes.

**Trial registration:**

Clinical Trials Registry India, CTRI202306054119, 19/06/2023. Retrospectively registered.

**Supplementary Information:**

The online version contains supplementary material available at 10.1186/s12887-025-06457-z.

## Introduction

Acute kidney injury in neonates is increasingly recognised as a significant contributor to morbidity and mortality in neonatal intensive care units particularly in low- and middle-income countries where diagnostic limitations and variable definitions have historically hindered accurate reporting and early detection. The introduction of standardised criteria such as the modified neonatal KDIGO framework has improved recognition; however, real-world Indian data remain limited, especially from Level IIIB NICUs with prospective design and post-discharge evaluation [1].

The reported incidence of neonatal AKI varies widely—from 4.2% to > 30%—depending on study design, gestational profile, diagnostic thresholds, and institutional care levels [2–4]. Indian studies applying KDIGO-based definitions have reported incidence rates between 4.2% and 8.8%, whereas the TINKER registry documented substantially higher figures among critically ill neonates requiring peritoneal dialysis [5]. These differences highlight how patient acuity, referral delay, and resource constraints influence the epidemiology of neonatal AKI across regions.

The pathogenesis of AKI in neonates is multifactorial. Sepsis, perinatal asphyxia, nephrotoxic medications, and haemodynamic instability are among the most frequently cited risk factors [5]. In a prospective study from North India, Gupta et al. identified multiple overlapping morbidities in preterm neonates with AKI, while an Iranian study by Momtaz et al. confirmed similar risk associations in a tertiary NICU setting [5, 6].

Referral patterns and NICU admission sources also influence AKI outcomes. In many Indian centres, a significant proportion of admissions are outborn neonates who face delays in diagnosis and stabilisation. A recent study by Gopalan and Hapani in a Level III NICU highlighted a significantly higher incidence and poorer outcomes among preterm outborn neonates with AKI compared to their inborn counterparts [7].

Despite several regional reports, few Indian studies have simultaneously evaluated AKI staging, nephrotoxin exposure, and short-term post-discharge somatic growth. Addressing these knowledge gaps, the present prospective study aimed to determine the incidence, clinical profile, and early growth outcomes of AKI among term and preterm neonates admitted to a Level IIIB NICU in South India, using modified neonatal KDIGO criteria and six-week anthropometric follow-up.

## Methodology

### Study design and setting

This prospective observational study was conducted in the Level IIIB Neonatal Intensive Care Unit (NICU) of a tertiary-care teaching hospital in South India from January 2023 to January 2024. The NICU admits both inborn and referred outborn neonates and provides advanced ventilatory, hemodynamic and renal-support facilities. The study protocol was approved by the Institutional Ethics Committee of Kasturba Medical College, Manipal Academy of Higher Education (IEC No. IEC2-309/2022), and written informed consent was obtained from parents or legal guardians prior to enrolment.

### Participants

All term and preterm neonates admitted during the study period were screened for eligibility. Neonates fulfilling the modified neonatal Kidney Disease Improving Global Outcomes (KDIGO) criteria for AKI were included. Those with major congenital anomalies, chromosomal disorders, incomplete biochemical data, or who were referred from other centers or died within 24 h of admission were excluded. Gestational age and birth-weight categories were assigned according to Fenton growth charts to identify preterm and small-for-gestational-age infants.

### Renal function testing and assessment criteria

Renal-function testing (RFT) was performed in 645 of 925 neonates (69.7%) based on predefined clinical triggers to avoid unnecessary sampling. The triggers included suspected or confirmed sepsis, shock or inotropic support, exposure to nephrotoxic drugs such as aminoglycosides or vancomycin, oliguria or progressive fluid overload, mechanical ventilation, perinatal asphyxia, congenital heart disease, postoperative state, and suspected congenital anomalies of kidney and urinary tract. Stable short-stay neonates without any of these conditions were not routinely tested.

### Definition and staging of AKI

Acute kidney injury was defined and staged using modified neonatal KDIGO criteria based on both serum-creatinine and urine-output parameters. Stage 1 was defined by a rise in serum creatinine of at least 0.3 mg/dL within 48 h or 1.5–1.9 times baseline; Stage 2 by an increase of two to 2.9 times baseline; and Stage 3 by an increase of three or more times baseline, an absolute value ≥ 2.5 mg/dL, or need for dialysis. Corresponding urine-output thresholds were < 1 mL/kg/h for Stage 1, < 0.5 mL/kg/h for Stage 2, and < 0.3 mL/kg/h for Stage 3 or anuria for 12 h. Baseline creatinine was defined as the lowest measured value after 48–72 h of life to minimise maternal influence, and urine output was measured by pre-weighed diaper or calibrated collection bag.

### Definitions of key variables

Definitions for key variables were standardised. Culture-negative sepsis referred to clinical sepsis with raised inflammatory markers but sterile blood culture. Shock was defined by prolonged capillary refill greater than three seconds and hypotension requiring fluid or inotropic therapy. Hypernatraemic dehydration referred to serum sodium ≥ 150 mmol/L with more than 10% weight loss, while nephrotoxin exposure indicated administration of at least one nephrotoxic medication during NICU stay. Outborn status denoted delivery outside the study centre with subsequent referral.

### Management protocol

All AKI cases were managed according to a uniform protocol emphasising fluid and electrolyte optimisation, strict avoidance of nephrotoxins, dose adjustment for renally excreted drugs, prompt correction of hypotension and hypovolaemia with isotonic fluids and vasoactive agents, and early institution of peritoneal dialysis for refractory hyperkalaemia, anuria exceeding 24 h, or progressive azotaemia. Nutritional support was individualised based on gestational age and renal tolerance.

### Data collection

Demographic, clinical and laboratory parameters were prospectively recorded using a pre-tested proforma. Daily serum-creatinine and electrolyte trends were monitored until recovery or discharge. Post-discharge anthropometric evaluation, including weight, length and head circumference, was performed at six weeks of corrected age to document early catch-up growth. Anthropometric measurements were performed by trained neonatal staff using standardized NICU procedures. Assessors were not informed of AKI staging at the time of measurement, although formal blinding was not implemented. Plans for continued six- and twelve-month follow-up focusing on renal function, blood pressure, and estimated glomerular filtration rate have been initiated as part of the ongoing longitudinal cohort.

### Statistical analysis

Data analysis was carried out using IBM SPSS Statistics version 26.0. Continuous variables were expressed as mean ± standard deviation or median (interquartile range) and compared using Student’s t test or Mann–Whitney U test. Categorical variables were expressed as number and percentage and compared using chi-square or Fisher’s exact test as appropriate. Variables with *p* < 0.20 on univariate analysis or deemed clinically significant were entered into multivariate logistic regression to identify independent predictors of Stage 3 AKI and mortality. Collinearity was excluded by variance-inflation-factor testing, and model adequacy was confirmed by Hosmer–Lemeshow goodness-of-fit (*p* = 0.61) and Nagelkerke R² (0.42). Model selection followed Akaike and Bayesian information criteria. Because the study was time-bound, a priori sample-size calculation was not feasible; however, precision analysis demonstrated an 8.2% AKI incidence with 95% confidence interval (6.2–10.5%) and at least ten events per variable, ensuring statistical reliability. A two-tailed p value < 0.05 was considered significant.

## Results

During the study period, 925 neonates were admitted to the Level IIIB NICU. Renal function testing (RFT) was performed in 645 (69.7%) neonates according to predefined clinical triggers. Fifty-four neonates initially met the modified neonatal KDIGO criteria for acute kidney injury (AKI); one was excluded because of early referral, leaving 53 eligible for final analysis. The overall incidence of AKI in the tested cohort was 8.2%. Characteristics of tested and untested neonates are presented in (Additional File 1).

Of the 53 neonates with AKI, 23 (43.4%) were preterm and 30 (56.6%) were term. Among preterm neonates, the largest subgroup was early preterm (10 of 23, 43%) and moderate-to-late preterm (9 of 23, 39%). In the term group, 25 of 30 (83%) were outborn compared with 9 of 23 (39%) in the preterm group. Most preterm neonates had a birth weight < 2500 g, with 56% (13 of 23) below 1500 g. Among term neonates, 90% (27 of 30) weighed > 2500 g. APGAR scores > 5 at 5 min were seen in 78% (18 of 23) of preterm and 83% (25 of 30) of term neonates. (Table 1) summarises baseline demographics.

**Table 1:** Baseline demographics in study population

Parameter	Preterm (*n* = 23)	Term (*n* = 30)
Gestational age		
< 28 weeks	4 (17%)	0
28–31 + 6 weeks	10 (43%)	0
32–36 + 6 weeks	9 (39%)	0
> 37 weeks	0	30
Place of delivery		
Outborn	9 (39%)	25 (83%)
Inborn	14 (61%)	5 (17%)
Sex		
Male	11 (47%)	18 (60%)
Female	12 (53%)	12 (40%)
Birth weight		
< 1000 g	6 (26%)	0
1000–1500 g	7 (30%)	0
1501–2500 g	8 (34%)	3 (10%)
> 2500 g	2 (8%)	27 (90%)
Mode of delivery		
VD	7 (30%)	16 (53%)
LSCS	16 (70%)	14 (47%)
Weight for GA		
AGA	16 (70%)	25 (83%)
SGA	7 (30%)	4 (13%)
LGA	0	1 (3%)
APGAR > 5 at 5 min	18 (78%)	25 (83%)
Resuscitation		
Basic	17 (73%)	21 (70%)
Advanced	6 (27%)	9 (30%)

All data are expressed as number (percentage) unless otherwise stated. *GA* gestational age, *AGA* appropriate for gestational age, *SGA* small for gestational age, *LGA* large for gestational age. Percentages are rounded to the nearest whole number and may not total 100% because of rounding

At presentation, 6 of 23 (26%) preterm and 9 of 30 (30%) term neonates required intubation. Cardiogenic shock was the predominant subtype (26% in both groups). Inotropic support was administered to 47% (11 of 23) of preterms and 66% (20 of 30) of terms, with dopamine used first-line and noradrenaline for refractory shock. Oliguria occurred more often in term neonates (11 of 30, 36%) than in preterms (5 of 23, 21%). Clinical characteristics are presented in (Table 2).

**Table 2:** Clinical profile in study population with AKI

Parameter	Preterm (*n* = 23)	Term (*n* = 30)
Bag & mask resuscitation at birth	7 (30%)	5 (16%)
Intubation at birth	6 (26%)	9 (30%)
Shock type		
Distributive	4 (17%)	3 (10%)
Hypovolemic	1 (4%)	5 (16%)
Cardiogenic	6 (26%)	8 (26%)
Obstructive	1 (4%)	3 (10%)
HIE stage		
Stage 1	1	1
Stage 2	0	4
Stage 3	0	4
Oliguria	5 (21%)	11 (36%)
Dehydration / Fever	3 (13%)	5 (16%)
Inotropes required	11 (47%)	20 (66%)
Significant weight loss	3 (13%)	5 (16%)

Percentages are shown for the proportion of neonates in each gestational subgroup. *HIE* hypoxic−ischaemic encephalopathy. “Shock” classification follows standard neonatal definitions (distributive, hypovolaemic, cardiogenic, and obstructive). Values represent the proportion affected within each gestational category

Sepsis (both culture-negative and culture-positive) remained the leading cause of AKI, contributing to 64% of all cases. Other frequent associations included congenital heart disease (53%), congenital anomalies of the kidney and urinary tract (CAKUT, 17%), and drug-induced nephrotoxicity (63%). Multiple concurrent aetiologies were common, and therefore percentages exceeded 100%. Hypernatraemic dehydration was observed in 8 (15%) neonates. Aminoglycoside exposure, predominantly amikacin, accounted for 62% of drug-related cases; vancomycin and diuretic use were less frequent. These findings are detailed in (Table 3).

**Table 3:** Aetiology contributing to AKI in study population

Parameter	Preterm (*n* = 23)	Term (*n* = 30)
Sepsis		
Culture – negative	6 (26%)	10 (36%)
Culture – positive	8 (34%)	7 (23%)
CHD		
Acyanotic	10 (60%)	15 (56%)
Cyanotic	1 (4%)	2 (6%)
CAKUT	6 (26%)	3 (10%)
NEC	2 (2b, 3a)	0
Drugs exposure		
Amikacin	16 (69%)	17 (56%)
Vancomycin	1 (4%)	2 (6%)
Diuretics	4 (17%)	3 (10%)
Antiviral (oseltamivir)	1 (4%)	0
Hypernatremic dehydration	3 (13%)	5 (16%)

Percentages exceed 100% because more than one aetiological factor was present in several neonates. *CHD* congenital heart disease, *CAKUT* congenital anomalies of the kidney and urinary tract, *NEC* necrotising enterocolitis. Drug exposure includes aminoglycosides (amikacin), glycopeptides (vancomycin), diuretics, and antiviral agents (oseltamivir). Culture−negative sepsis refers to clinical sepsis fulfilling modified CDC criteria with negative blood culture. Data are expressed as n (%) unless otherwise specified

Of the 53 neonates, 27 (51%) survived and 26 (49%) died. Mortality increased with AKI severity: 15 of 36 (42%) in Stage 1, 2 of 7 (29%) in Stage 2, and 9 of 10 (90%) in Stage 3 (*p* = 0.032). Stage 3 AKI had the highest lethality. Full outcome stratification is shown in (Table 4).

**Table 4:** Outcomes based on staging of AKI*

Stage	Survived(*n* = 27)	Death(*n* = 26)
1(*n* = 36)	21(58%)	15(42%)
2(*n* = 7)	5(71%)	2(29%)
3(*n* = 10)	1(10%)	9(90%)

AKI staging defined by modified neonatal KDIGO criteria. Percentages represent survival and mortality within each stage category

Post-discharge follow-up was achieved for all 27 survivors at 6 weeks. The greatest weight gain was observed among preterm small-for-gestational-age (SGA) neonates (38.5 g/day; 5.0 cm/month in length), whereas term SGA neonates had the lowest gains (22.1 g/day; 1.8 cm/month). Head-circumference gains were similar across groups. Growth recovery data are presented in (Table 5).

**Table 5:** Growth pattern at 6 weeks of discharge in survivors of AKI*

	Term(*n* = 14)		Preterm(*n* = 12)	
	SGA(*n* = 3)	AGA(*n* = 11)	SGA(*n* = 2)	AGA(*n* = 10)
Gain in Weight(g/day)	22.1	28.1	38.5	21.2
Gain in Length (cm/month)	1.8	2.5	5	2.6
Gain in HC (cm/month)	1.6	1.4	1.8	1.6

Growth parameters measured at 6−week follow−up visit. Weight gain is presented as grams per day; length and head−circumference (HC) gains are expressed as centimetres per month. SGA = small for gestational age; AGA = appropriate for gestational age

**Fig. 1:**
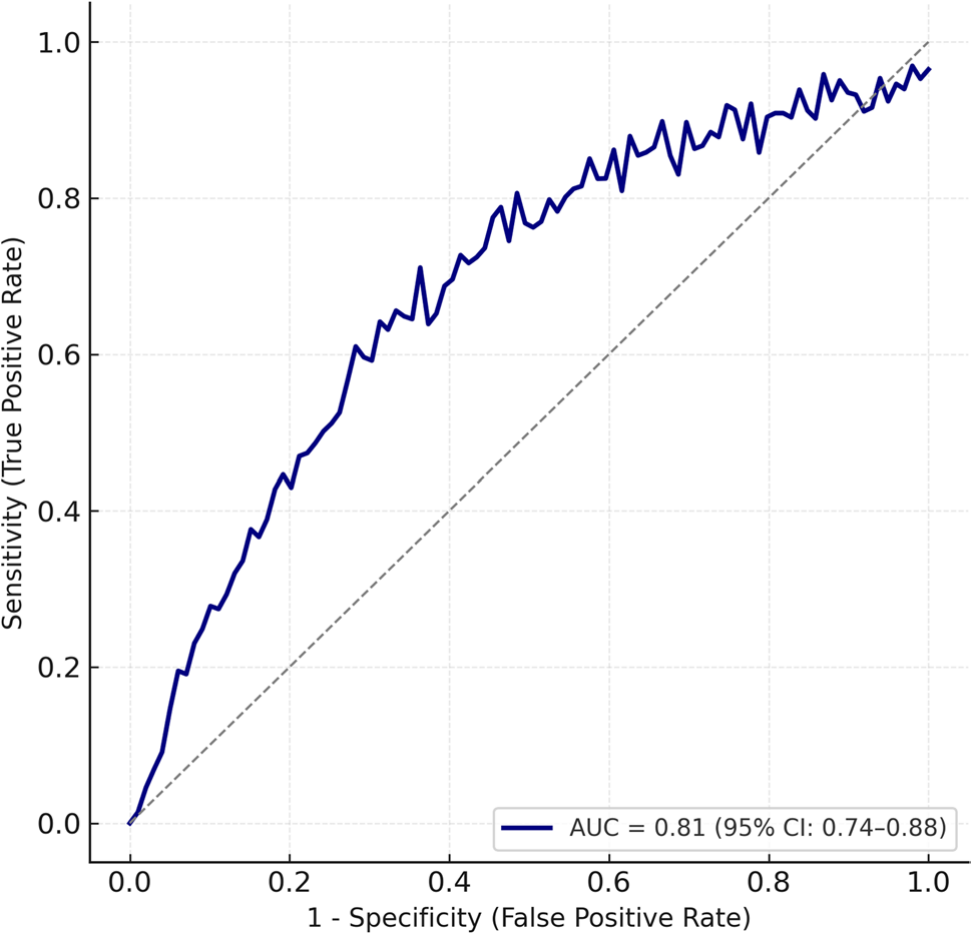
Receiver Operating Characteristic (ROC) curve for prediction of severe AKI or mortality in neonates

Univariate logistic regression identified outborn status, sepsis, inotrope requirement, and oliguria as significant predictors of severe AKI or death (Additional File 1). In the multivariate model (Table 6), outborn status (aOR 3.15, 95% CI 1.02–9.74; *p* = 0.046), inotropic use (aOR 2.78, 95% CI 1.09–7.13; *p* = 0.032), and sepsis (aOR 2.96, 95% CI 1.08–8.12; *p* = 0.035) independently predicted Stage 3 AKI or mortality, while oliguria lost statistical significance (aOR 1.45, 95% CI 0.51–4.16; *p* = 0.482). Model calibration was good (Hosmer–Lemeshow *p* = 0.61) and discrimination fair (Nagelkerke R² = 0.42). The corresponding ROC curve yielded an AUC of 0.81 (95% CI 0.74–0.88), confirming satisfactory predictive performance (Fig. 1).

**Table 6:** Multivariate logistic regression for predictors of stage 3 AKI or mortality

Variable	Adjusted Odds Ratio (aOR)	95% CI	*p*-value
Outborn status	3.15	1.02–9.74	0.046
Inotropic use	2.78	1.09–7.13	0.032
Sepsis	2.96	1.08–8.12	0.035
Oliguria	1.45	0.51–4.16	0.482

Variables with *p* < 0.20 in univariate analysis (Supplement S1) were included in the multivariate model. *aOR* adjusted odds ratio, *CI* confidence interval. Model diagnostics: Hosmer–Lemeshow *p* = 0.61; Nagelkerke R² = 0.42

Cross-tabulation analyses reinforced these associations. Mortality among outborn neonates was 58.8% (20 of 34) compared with 31.6% (6 of 19) among inborn neonates (Table 1). Neonates with sepsis had 58.1% mortality (18 of 31), identical to that observed among those receiving inotropes (18 of 31) (Tables 2 and 3). Among neonates presenting with oliguria, 81.2% (13 of 16) died versus 43.4% (10 of 37) without oliguria (Table 2). Stage 3 AKI had the highest mortality (90%), consistent with regression findings (Table 4).

Subgroup analysis of post-discharge growth revealed that infants recovering from Stage 1 AKI had the highest average weight gain (28.4 g/day) compared with 18.5 g/day after Stage 3 AKI. Length gain followed the same pattern (2.8, 2.2, and 1.6 cm/month for Stages 1–3 respectively), paralleling head-circumference increments (1.6, 1.4, and 1.2 cm/month). The frequency of nephrotoxic drug exposure was greatest in Stage 2 (86%), followed by Stage 3 (60%) and Stage 1 (58%), suggesting a possible link between cumulative nephrotoxin burden and AKI severity (Table 3).

## Discussion

Acute kidney injury (AKI) in neonates is increasingly recognized as a major contributor to neonatal morbidity and mortality, with growing interest in risk stratification, staging, and long-term outcomes. In this Level IIIB NICU cohort study, the overall incidence of AKI among tested neonates was 8.2%, consistent with previous Indian studies (5–9%) but substantially lower than the global multicentric AWAKEN study, which reported a 30% incidence using similar KDIGO criteria [8]. This difference may reflect referral population variations, the exclusion of early discharges, and stricter biochemical confirmation in our methodology. The inclusion of only 645 of 925 NICU admissions for renal function testing introduces possible selection bias; however, Additional File 1 demonstrates that untested neonates were typically low-risk, short-stay admissions, supporting the representativeness of the tested cohort.

The study findings reaffirm the relatively higher burden of AKI among term neonates (57%) compared to preterms—a pattern similar to that observed by Lee et al., who reported substantial AKI occurrence in term infants with sepsis or hypoxia [9]. In this study, 83% of term neonates were outborn, often presenting with advanced illness, haemodynamic instability, and greater nephrotoxin exposure—factors known to adversely influence renal outcomes. Outborn status emerged as an independent predictor of severe AKI and mortality (aOR 3.15), highlighting systemic differences in perinatal stabilization and referral pathways.

Sepsis and shock were the dominant aetiologies of AKI, collectively accounting for nearly 60% of cases. Mathur et al. similarly identified sepsis-associated hypotension and hypoperfusion as key drivers of renal injury [10]. Kungwani et al. also reported sepsis as a leading cause in Indian NICUs [11]. Cardiogenic shock was frequent in this study series (26% across gestational subgroups), underscoring the pathophysiologic link between myocardial dysfunction and renal hypoperfusion in neonatal AKI.

Inotropic support was required in 58.5% of affected neonates, confirming the overlap between circulatory failure and renal dysfunction. This proportion aligns with Gopal’s study on perinatal asphyxia, which demonstrated that both hypoxic insult and inotrope dependency were strong AKI correlates [12]. Multivariate regression in the study cohort confirmed inotrope use (aOR 2.78) as an independent predictor of severe AKI or death, reinforcing its prognostic significance. These findings are supported by similar associations reported in Indian and global literature [12, 13].

Although oliguria was documented in only 30% of cases, it carried a disproportionately high mortality risk (81.2%). This aligns with Durga and Rudrappa, who noted oliguria as a late but ominous indicator of renal decompensation in septic neonates [13]. Stage 3 AKI was associated with 90% mortality—higher than previously reported by Garg et al. and Criss et al., where Stage 3 AKI and NEC were the principal determinants of poor outcome [14, 15]. This steep gradient across KDIGO stages validates the staging system as both a diagnostic and prognostic tool within Indian NICUs.

Nephrotoxic drug exposure, mainly to aminoglycosides, was documented in 63% of the study cohort. Stage 2 AKI had the highest nephrotoxin burden (86%), followed by Stage 3 (60%). These results are consistent with Viswanathan et al., who highlighted aminoglycoside-associated AKI in extremely low birth weight infants [16]. Although drug exposure was not an independent mortality predictor in regression model, cumulative nephrotoxin burden likely contributes to progression, emphasising the need for structured neonatal nephrotoxin stewardship programs.

A key strength of this study is the prospective design with defined KDIGO criteria and follow-up anthropometry. The 6-week follow-up revealed variable post-AKI growth recovery, with preterm SGA infants showing remarkable catch-up growth (38.5 g/day, 5.0 cm/month) compared to term SGA infants (22.1 g/day, 1.8 cm/month). These findings concur with Joshi et al., who demonstrated early anthropometric recovery following AKI, particularly in preterms with adequate nutritional support [16]. However, this short follow-up remains a limitation; ongoing 6- and 12-month evaluations are planned to include blood pressure, GFR estimation, and renal ultrasound to assess long-term outcomes such as hypertension and chronic kidney disease.

Sample size and power were constrained by the single-centre design and limited events per variable; nonetheless, precision-based calculations ensured sufficient statistical reliability (95% CI 6.2–10.5; EPV ≥ 10). The logistic model demonstrated acceptable calibration (Hosmer–Lemeshow *p* = 0.61) and good discrimination (AUC 0.81, 95% CI 0.74–0.88). The overall model performance supports the robustness of identified predictors—outborn status, sepsis, and inotrope use—as clinically relevant risk markers.

Current study results complement recent data by Gopalan and Hapani [7], who reported a 13.3% AKI incidence in a comparable Indian NICU cohort. The addition of post-discharge anthropometry and multivariate risk modelling makes our study distinct, offering more granular insight into modifiable clinical predictors. The slightly lower incidence (8.2% vs. 13.3%) likely reflects referral filtering and differences in sampling frequency. Together, these findings strengthen the evidence base for risk-based RFT screening and integrated renal monitoring in Indian NICUs.

### Strengths and limitations

Prospective design, use of standardized neonatal KDIGO definitions, and incorporation of post-discharge anthropometric follow-up lend robustness and granularity to the present data. Nonetheless, certain limitations must be acknowledged. Being a single-center study, the findings may not be generalizable to wider NICU populations. Although 645 of 925 neonates underwent renal function testing, selection bias cannot be completely excluded; however, untested neonates were predominantly low-risk, short-stay admissions. The sample size was modest, but the study achieved acceptable statistical precision (95% CI 6.2–10.5) and adequate model stability with events-per-variable ≥ 10. Urinary biomarkers such as NGAL and cystatin C were not assessed, which may have enhanced early AKI detection. Additionally, serum creatinine variability within the first 48 h could not be consistently captured due to intermittent sampling. Despite these limitations, the internal consistency of KDIGO staging, risk-factor stratification, and post-AKI follow-up strengthens the validity of the observations. The multivariate model demonstrated good calibration (Hosmer–Lemeshow *p* = 0.61) and discrimination (AUC 0.81), confirming the reliability of the derived predictors for severe AKI and mortality.

## Conclusion

Acute kidney injury in neonates remains a significant contributor to morbidity and mortality in intensive care. In this Level IIIB NICU cohort, the incidence of AKI was 8.2%, with sepsis, outborn status, and inotropic dependency emerging as independent predictors of severe AKI or death. The KDIGO staging system reliably stratified disease severity, as reflected by the strong predictive accuracy of the multivariate model. Mortality reached 90% among Stage 3 AKI cases, underscoring the urgent need for early recognition and hemodynamic optimization. Post-AKI survivors demonstrated early catch-up growth, particularly among preterm SGA infants, suggesting that nutritional and renal recovery can progress in parallel when follow-up is structured. These findings highlight the importance of risk-based renal function monitoring, nephrotoxin stewardship, and longitudinal surveillance—including blood pressure, eGFR, and renal ultrasound—to mitigate long-term sequelae of neonatal AKI.

## Supplementary Information

Supplementary Material 1.

## Data Availability

The raw data supporting the conclusions of this article will be made available by the authors without undue reservation.

## Abbreviations

AKI: Acute Kidney Injury
NICU: Neonatal Intensive Care Unit
KDIGO: Kidney Disease: Improving Global Outcomes
RFT: Renal Function Test
SGA: Small for Gestational Age
CAKUT: Congenital Anomalies of the Kidney and Urinary Tract
GFR / eGFR: Glomerular Filtration Rate / estimated Glomerular Filtration Rate
AUC: Area Under the Curve
ROC: Receiver Operating Characteristic
OR / aOR: Odds Ratio / Adjusted Odds Ratio
CI: Confidence Interval
IQR: Interquartile Range
SD: Standard Deviation
NEC: Necrotizing Enterocolitis
NGAL: Neutrophil Gelatinase–Associated Lipocalin
MIN: Minutes (Apgar references)
CTRI: Clinical Trials Registry of India
IEC: Institutional Ethics Committee
MAHE: Manipal Academy of Higher Education
CA: Corrected Age
IVF: Intravenous Fluid
PD: Peritoneal Dialysis
VIF: Variance Inflation Factor
ELBW: Extremely Low Birth Weight
LBW: Low Birth Weight
CPR: Capillary Refill
HR: Heart Rate
BP: Blood Pressure

## References

[1] Gallo D, de Bijl-Marcus KA, Alderliesten T, Lilien M, Groenendaal F. Early acute kidney injury in preterm and term neonates: incidence, outcome, and associated clinical features. Neonatology. 2021;118(2):174–9. 10.1159/000514376. 33780939 10.1159/000513666

[2] Bansal SC, Nimbalkar AS, Kungwani AR, Patel DV, Sethi AR, Nimbalkar SM. Clinical profile and outcome of newborns with acute kidney injury in level 3 neonatal unit in Western India. J Clin Diagn Res. 2017;11(4):SC01–4. 10.7860/JCDR/2017/24851.9617. 28511469 10.7860/JCDR/2017/23398.9327 PMC5427395

[3] Kalita D, Rahman M, Barman D, Hussain S. Aetiological profile and outcomes of acute kidney injury among neonates admitted in a neonatal intensive care unit at a tertiary care hospital, Northeast india: a prospective cohort study. J Clin Diagn Res. 2023;17(11):SC10–2. 10.7860/JCDR/2023/62692.17687.

[4] Sethi SK, Wazir S, Sahoo J, Agrawal G, Bajaj N, Gupta NP, et al. Risk factors and outcomes of neonates with acute kidney injury needing peritoneal dialysis: results from the prospective TINKER (The Indian PCRRT-ICONIC neonatal kidney educational Registry) study. Perit Dial Int. 2022;42(5):460–9. 10.1177/08968608221079345. 35574693 10.1177/08968608221091023

[5] Gupta S, Gaur BK, Jain R, Singh RR. Incidence, risk factors, and outcomes of acute kidney injury in preterm neonates hospitalized in the neonatology unit, North india: a single-center experience. Saudi J Kidney Dis Transpl. 2023;34(6):592–601. 10.4103/sjkdt.sjkdt_264_23. 38725209 10.4103/sjkdt.sjkdt_264_23

[6] Momtaz HE, Sabzehei MK, Rasuli B, Torabian S. The main etiologies of acute kidney injury in the newborns hospitalized in the neonatal intensive care unit. J Clin Neonatol. 2014;3(2):99–102. 10.4103/2249-4847.134682. 25024976 10.4103/2249-4847.134691 PMC4089136

[7] Gopalan D, Hapani PT. Clinical profile and outcome of acute kidney injury in preterm neonates in a level three neonatal intensive care unit. Int J Contemp Pediatr. 2024;11(2). 10.18203/2349-3291.ijcp20240091.

[8] Jetton JG, Boohaker LJ, Sethi SK, Wazir S, Rohatgi S, Soranno DE, et al. Incidence and outcomes of neonatal acute kidney injury (AWAKEN): a multicentre, multinational, observational cohort study. Lancet Child Adolesc Health. 2017;1(3):184–194. 10.1016/S2352-4642(17)30069-X 10.1016/S2352-4642(17)30069-X. 29732396 10.1016/S2352-4642(17)30069-X PMC5933049

[9] Lee C, Chan W, Lai Y, Hsu H, Wu W, Lim H, et al. Incidence and outcomes of acute kidney injury in extremely-low-birth-weight infants. PLoS ONE. 2017;12(11):e0187764. 10.1371/journal.pone.0187764. 29108006 10.1371/journal.pone.0187764 PMC5673227

[10] Mathur NB, Agarwal HS, Maria A. Acute renal failure in neonatal sepsis. Indian J Pediatr. 2006;73(6):499–502. 10.1007/BF02759894. 16816511 10.1007/BF02759894

[11] Kungwani A, Nimbalkar A, Sethi A, et al. 1324 clinical profile and outcome of newborns with acute kidney injury in a level 3 neonatal unit in Western India. Arch Dis Child. 2012;97(Suppl 2):A377. 10.1136/archdischild-2012-302724.1324. 10.7860/JCDR/2017/23398.9327 PMC5427395 28511469

[12] Gopal G. Acute kidney injury (AKI) in perinatal asphyxia. Indian J Pharm Biol Res. 2014;2(2):60.

[13] Durga D, Rudrappa S. Clinical profile and outcome of acute kidney injury in neonatal sepsis in a tertiary care centre. Int J Contemp Pediatr. 2017;4(2):635–8. 10.18203/2349-3291.ijcp20170968.

[14] Garg PM, Britt AB, Ansari MA, Sobisek S, Block DK, Paschal JL, et al. Severe acute kidney injury in neonates with necrotizing enterocolitis: risk factors and outcomes. Pediatr Res. 2021;90(3):642–9. 10.1038/s41390-021-01544-2. 33446918 10.1038/s41390-020-01320-6 PMC8277891

[15] Criss CN, Selewski DT, Sunkara B, et al. Acute kidney injury in necrotizing Enterocolitis predicts mortality. Pediatr Nephrol. 2018;33(3):503–10. 10.1007/s00467-017-3824-3. 28983789 10.1007/s00467-017-3809-y

[16] Viswanathan S, Manyam B, Azhibekov T, Mhanna MJ. Risk factors associated with acute kidney injury in extremely low birth weight infants. Pediatr Nephrol. 2012;27(2):303–11. 10.1007/s00467-011-1964-1. 21809002 10.1007/s00467-011-1977-8

