# Supplementary material for: Prognostic value of modified KDIGO staging for acute kidney injury in neonates: a prospective observational study in a level IIIB NICU
Source: BMC Pediatr. 2026 Jan 20;26:169. doi: 10.1186/s12887-025-06457-z (PMC12951903; doi:10.1186/s12887-025-06457-z)
Supplement: Supplementary file 1 — Supplementary Material 1. [file 12887_2025_6457_MOESM1_ESM.docx]

**Supplementary Table S0. Comparison of neonates who underwent renal function testing vs. those not tested (n = 925)**

| **Parameter** | **RFT Done (n = 645)** | **RFT Not Done (n = 280)** | **p-value** |
| --- | --- | --- | --- |
| Outborn delivery | 54 % | 38 % | 0.018 * |
| Preterm (< 37 weeks) | 46 % | 29 % | 0.011 * |
| Male sex | 58 % | 56 % | 0.72 |
| Sepsis (clinical ± culture) | 37 % | 11 % | < 0.001 * |
| Inotropic support | 21 % | 3 % | < 0.001 * |
| Ventilation required | 27 % | 9 % | < 0.001 * |
| Mortality | 8.4 % | 1.8 % | < 0.001 * |

***^Selection bias analysis showing higher illness acuity among tested neonates; p-values < 0.05 mark^*^ed*. Comparison between neonates who underwent renal-function testing and those who did not. p-values < 0.05 were considered statistically significant. Differences reflect illness-severity selection bias; statistical tests = χ² or Fisher’s exact as appropriate.^**

**Supplementary Table S1. Univariate logistic regression for predictors of mortality among neonates with AKI (n = 53)**

| **Variable** | **Unadjusted OR** | **95 % CI** | **p-value** |
| --- | --- | --- | --- |
| Outborn status | 3.42 | 1.25 – 9.32 | 0.017 * |
| Sepsis | 3.08 | 1.13 – 8.36 | 0.028 * |
| Inotropic use | 2.94 | 1.10 – 7.87 | 0.032 * |
| Oliguria | 1.89 | 0.69 – 5.14 | 0.22 |
| Stage 3 AKI | 8.60 | 1.80 – 41.01 | 0.006 * |
| NEC | 2.20 | 0.58 – 8.26 | 0.25 |
| CHD | 1.62 | 0.60 – 4.38 | 0.34 |

**^OR = odds ratio; CI = confidence interval. Variables with p < 0.20 entered the multivariate logistic model shown in Table 6. p-values < 0.05 were considered statistically significant.^**
